# Diagnosis of nasal bone fractures on plain radiographs via convolutional neural networks

**DOI:** 10.1038/s41598-022-26161-7

**Published:** 2022-12-13

**Authors:** Yoonho Nam, Yangsean Choi, Junghwa Kang, Minkook Seo, Soo Jin Heo, Min Kyoung Lee

**Affiliations:** 1 Division of Biomedical Engineering, Hankuk University of Foreign Studies, Yongin-si, Gyeonggi‐do Republic of Korea; 2 Department of Radiology, Seoul St. Mary’s Hospital, College of Medicine, The Catholic University of Korea, Seoul, Republic of Korea; 3 Department of Radiology, Yeouido St. Mary’s Hospital, College of Medicine, The Catholic University of Korea, Seoul, Republic of Korea

**Keywords:** Computational biology and bioinformatics, Medical research

## Abstract

This study aimed to assess the performance of deep learning (DL) algorithms in the diagnosis of nasal bone fractures on radiographs and compare it with that of experienced radiologists. In this retrospective study, 6713 patients whose nasal radiographs were examined for suspected nasal bone fractures between January 2009 and October 2020 were assessed. Our dataset was randomly split into training (n = 4325), validation (n = 481), and internal test (n = 1250) sets; a separate external dataset (n = 102) was used. The area under the receiver operating characteristic curve (AUC), sensitivity, and specificity of the DL algorithm and the two radiologists were compared. The AUCs of the DL algorithm for the internal and external test sets were 0.85 (95% CI, 0.83–0.86) and 0.86 (95% CI, 0.78–0.93), respectively, and those of the two radiologists for the external test set were 0.80 (95% CI, 0.73–0.87) and 0.75 (95% CI, 0.68–0.82). The DL algorithm therefore significantly exceeded radiologist 2 (*P* = 0.021) but did not significantly differ from radiologist 1 (*P* = 0.142). The sensitivity and specificity of the DL algorithm were 83.1% (95% CI, 71.2–93.2%) and 83.7% (95% CI, 69.8–93.0%), respectively. Our DL algorithm performs comparably to experienced radiologists in diagnosing nasal bone fractures on radiographs.

## Introduction

Nasal bone fractures are the most common fractures of the facial bones, accounting for up to 50% of all incidences^1^. Moreover, they represent the third most common bone fracture overall^2^. The anatomy of the nasal bone—a thin, protruded structure of the face—makes it especially vulnerable to fractures. Nasal bone fractures have various causes, including sports injuries, physical fights, traffic accidents, and falls^3^. Complications of nasal bone fractures vary in severity from mild forms, including nasal septal deviation, nasal obstruction, and olfactory disturbances, to more severe complications, such as cerebrospinal fluid rhinorrhea^4^. Careful physical, clinical, and radiographic examinations are primarily used to diagnose nasal bone fractures^5^.

Radiological modalities for diagnosing nasal bone fractures include high-resolution computed tomography (CT), plain radiography, and ultrasonography. Among these, CT shows the highest diagnostic accuracy and was found to also be superior in detecting other associated injuries^6^. However, plain nasal radiography still shows substantial reliability^1^, with a sensitivity of approximately 80%^7^. Considering the benefits of low radiation exposure as well as its cost-effectiveness and accessibility, plain radiography remains the initial diagnostic tool for screening simple nasal bone fractures.

Deep learning (DL) is a specialized subset of machine learning, a multilayered information processing technique. The abilities of DL systems include self-extraction of data from raw input and self-learning, and DL-based applications are emerging in various fields of medicine^8^. In recent studies, a specific subcategory of DL, convolutional neural networks (CNNs), have shown reliable and accurate performance in various radiological subspecialties; they are thus expected to show significant potential in improving patient outcomes in the future^9^. In medical imaging, DL has been applied in the diagnosis, classification, and prediction of outcomes or survival from a variety of diseases; applications include, for example, fatty liver disease risk stratification from CT images, the diagnosis of prostate cancer from MRI, and the prediction of acute respiratory disease prognosis using CT^9^.

As conventional radiography is used in various medical subspecialties, a number of studies have reported on DL algorithms that detect abnormalities on radiographs and verified their diagnostic performance compared to that of radiologists. For instance, DL algorithms showed outstanding results in chest radiograph interpretation^10^, the detection of hip fractures^11^, and the diagnosis of moyamoya disease^12^, paranasal sinusitis^13,14^, proximal humerus fractures^15^, knee osteoarthritis^16^, pediatric skull fractures^17^, and developmental dysplasia of the hip^18^. Furthermore, a recent study explored the potential of DL in diagnosing nasal fractures in CT scans^19^. However, the DL-based detection of nasal bone fractures using conventional radiography has not been investigated. Therefore, here, we aimed to design a CNN-based algorithm to detect nasal bone fractures on plain radiographs and compare its diagnostic performance with that of radiologists.

## Results

### Patients

The baseline characteristics of the study cohort are summarized in Table 1. The training, validation, and internal test cohorts consisted of 4325, 481, and 1250 patients, respectively (mean age, 44–46 years; men, 58.5–68.2%; fractures, 45.2–46.2%). The external test cohort included 102 patients (mean age, 46 years; men, 52%; fractures, 57.8%). There were no significant differences among the cohorts in mean age (*P* = 0.101) or proportion of fractures (*P* = 0.06). There were significantly more men in the training and validation cohorts (67.6–68.2%) than in the internal and external test sets (52–58.5%) (*P* < 0.001).

**Table 1:** Baseline characteristics of patients.

Characteristics	Training set (n = 4325)	Validation set (n = 481)	Internal test set (n = 1250)	External test set (n = 102)	*P*-value*
Age, mean ± SD	44 ± 18	44 ± 18	46 ± 20	46 ± 19	0.101
**Sex, n (%)**					< 0.001
Men	2950 (68.2)	325 (67.6)	731 (58.5)	53 (52)	
Women	1375 (31.8)	156 (32.4)	519 (41.5)	49 (48)	
**Label, n (%)**					0.06
Fracture	1996 (46.2)	222 (46.2)	565 (45.2)	59 (57.8)	
Normal	2329 (53.8)	259 (53.8)	685 (54.8)	43 (42.2)	

*SD* standard deviation.

*P-values were calculated using one-way ANOVA or Pearson’s Chi-squared test, where appropriate.

### Diagnostic performance of the deep learning model and radiologists

**Figure 1:**
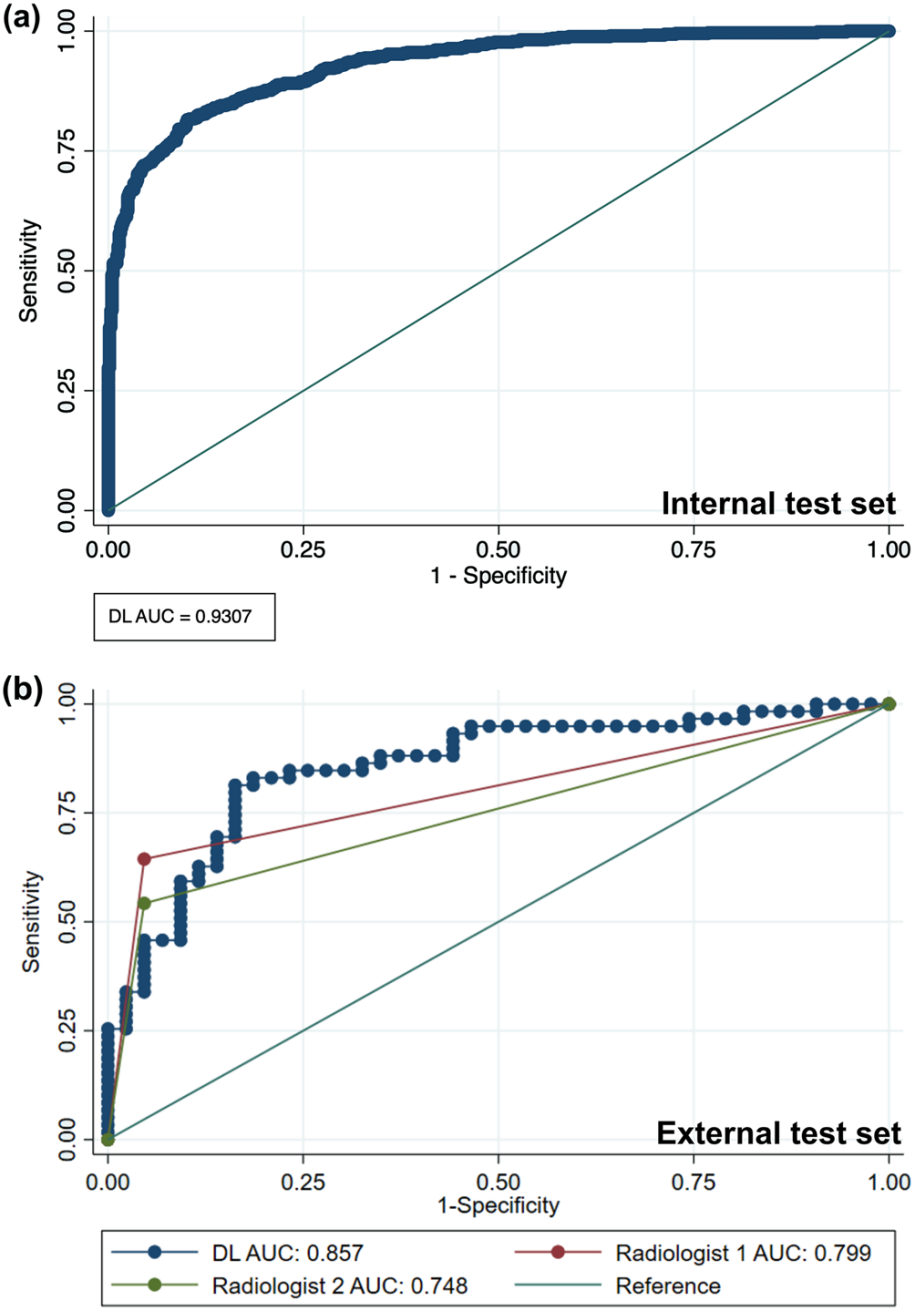
HYPERLINK "sps:id::fig1||locator::gr1||MediaObject::0" Receiver operating characteristics curves of the deep learning algorithm in the (**a**) internal test set and (**b**) external test set with radiologists.

On the internal test set, the DL model demonstrated excellent diagnostic performance with an AUC of 0.931 (95% CI, 0.915–0.944), sensitivity of 82.2% (95% CI, 78.3–86.5%), specificity of 89.6% (95% CI, 85.2–92.4%), and accuracy of 85.9% (95% CI, 84–87.8%) (Fig. 1a).

The diagnostic performance measures of the DL model and the two radiologists on the external test set are listed in Table 2. The DL model showed an AUC of 0.857 (95% CI, 0.782–0.931), sensitivity of 83.1% (95% CI, 71.2–93.2%), specificity of 83.7% (95% CI, 69.8–93%), and accuracy of 83.3% (95% CI, 75.5–90.2%). The AUCs of radiologists 1 (blinded, with 9 years of experience) and 2 (blinded, with 6 years of experience) were 0.799 (95% CI, 0.729–0.868) and 0.749 (95% CI, 0.676–0.819), respectively. The AUCs of the two radiologists did not significantly differ (*P* = 0.172). The inter-rater agreement between the two radiologists was substantial (Cohen κ coefficient, 0.67), whereas it was fair to moderate between the radiologists and the DL model (Cohen κ coefficient, 0.28–0.41).

**Table 2:** Diagnostic performance of deep learning model and radiologists.

	Accuracy (%)	Sensitivity (%)	Specificity (%)	AUC	*P*-value*
Deep learning model	83.3 (75.5–90.2)	83.1 (71.2–93.2)	83.7 (69.8–93.0)	0.857 (0.782–0.931)	
Radiologist 1	77.5 (69.6–84.3)	64.4 (52.5–76.3)	95.3 (88.4–100)	0.799 (0.729–0.868)	0.142
Radiologist 2	71.6 (63.7–79.4)	54.2 (42.4–67.8)	95.3 (88.4–100)	0.749 (0.676–0.819)	0.021

*AUC* area under the receiver operating characteristics curve.

*P-values calculated using DeLong’s method for comparing AUCs between the deep learning model and the two radiologists.

Numbers in parentheses indicate 95% confidence intervals.

### Comparison of diagnostic performance measures

**Figure 2:**
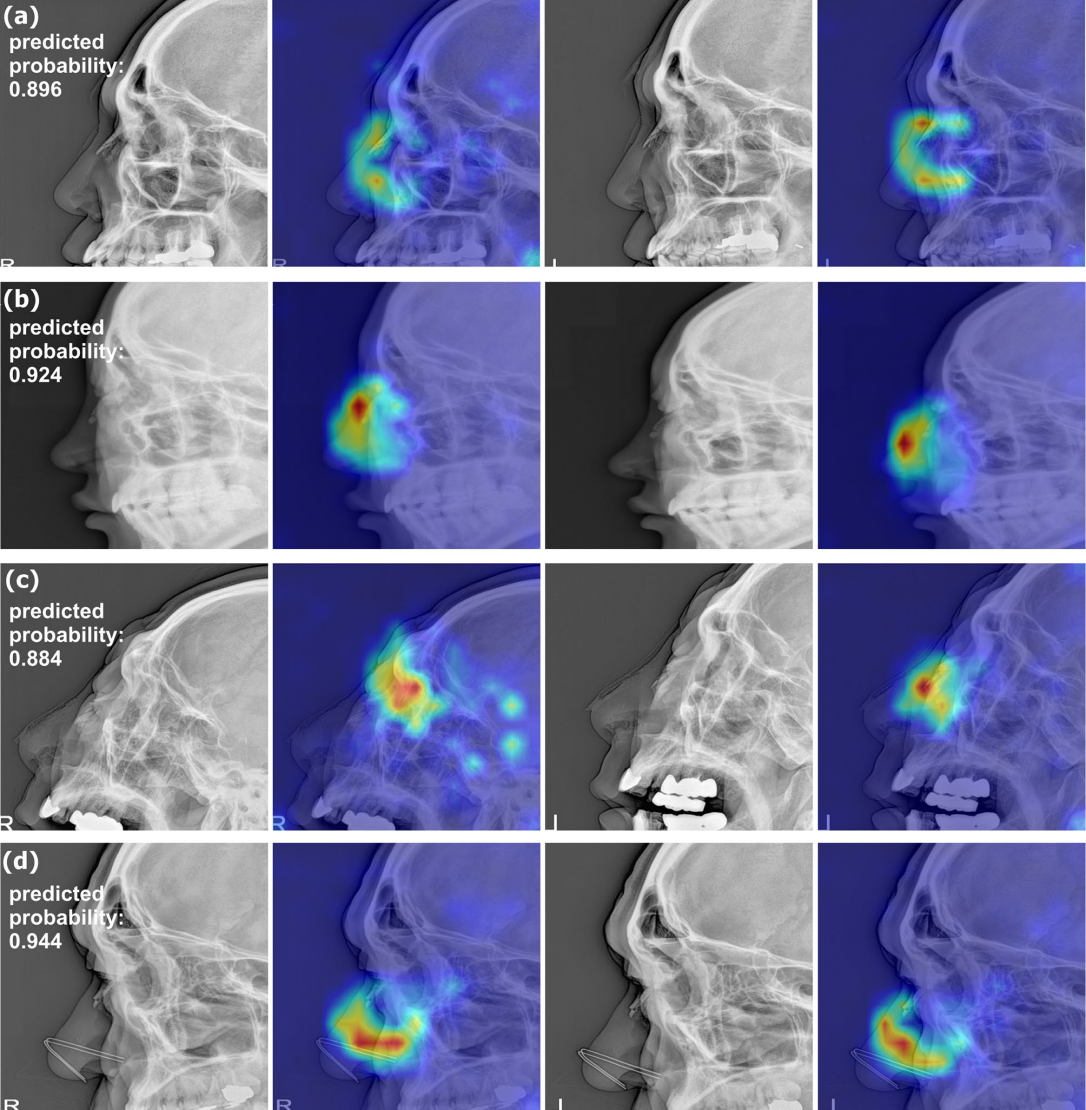
Example images of bilateral nasal bone radiographs overlaid with Grad-CAM heatmaps. The DL model and radiologists all correctly identified the nasal bone fractures. The images (**a**) and (**b**) show typical nasal bone fractures with fracture lines; images (**c**) and (**d**) show comminuted fractures with skin folds (**c**) and a metallic nose wire from a facial mask (**d**). The predicted probabilities of fractures by the DL model are shown for each patient.

**Figure 3:**
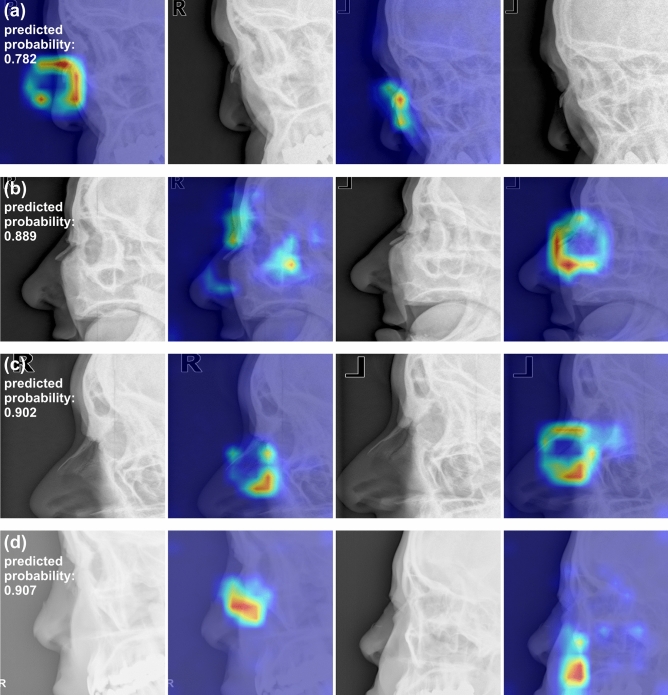
Examples of false positive images (normal nasal bone radiographs incorrectly diagnosed as fractures by the DL model). (**a**) The heatmap focuses on the angulated nasal bone in the left lateral view (right); (**b**) the heatmap focuses on nasal area narrowly separated from adjacent facial bones in the left lateral view (right); (**c**) the heatmap focuses on linear radiolucent line besides the nasal bone in the left lateral view (right); (**d**) the nasal bone is small and thin in the right lateral view (left) and faintly visible in the left lateral view (right). The predicted probabilities of fractures by the DL model are shown for each patient.

The AUC of the DL model was significantly higher than that of radiologist 2 (0.857 vs. 0.749; *P* = 0.021) but not significantly different from that of radiologist 1 (0.857 vs. 0.799; *P* = 0.142). The ROC curves of the DL model and those of the two radiologists are depicted in Fig. 1b. Example radiographs of correctly diagnosed nasal bone fractures with Grad-CAM heatmaps are shown in Fig. 2. The nasal bone area was properly highlighted in the heatmap in both views for most patients (n = 90). For nine of the remaining 12 subjects, the nasal bone was highlighted in only one view while the nasal bone was not highlighted in both views in three patients. Additionally, examples of normal nasal radiographs incorrectly diagnosed as fractures by the DL model are shown in Fig. 3.

## Discussion

The current study trained and validated a CNN-based DL model for the diagnosis of nasal bone fractures using plain bilateral radiographs. The DL model demonstrated excellent diagnostic performance on both the internal (AUC: 0.931) and external (AUC: 0.857) test sets. Furthermore, the DL model showed a diagnostic performance comparable to that of experienced radiologists (AUC: 0.749–0.799).

With regard to more recent studies comparing the diagnostic performance of DL and radiologists on conventional radiographs, DL models have been shown to outperform radiologists in diagnosing maxillary^13^ and paranasal sinusitis^14^ and have demonstrated comparable diagnostic performance for pediatric supracondylar fractures^20^ and developmental dysplasia of the hip^18^ as well as general chest radiograph interpretation^10^. However, it is important to note that the DL model’s comparable performance to radiologists does not lessen the need for critical appraisal by human practitioners, including a comprehensive review of a patient’s clinical information; rather, it should be seen as a complimentary diagnostic aid.

A previous study found that only 82% of nasal bone fractures can be identified on plain radiographs^1^. In this study, the DL model had a sensitivity of 83.1%, indicating an almost perfect diagnostic performance for this imaging modality. While CT images with thin slice thickness reconstruction are the imaging modality of choice for diagnosing nasal bone fractures^21^, conventional radiographs have advantages such as lower radiation exposure, fast image acquisition, and cost-effectiveness. Although conventional radiography is not the most accurate imaging modality for diagnosing nasal bone fractures, DL-assisted radiography would help expedite the diagnosis of nasal bone fractures and address resource scarcity in clinical practice. However, the definite diagnosis of nasal bone fractures depends on both CT and conventional radiographs owing to the inherently limited diagnostic capability of conventional radiographs.

The main strength of the current study is that our DL model was trained on well-balanced real-world clinical datasets, and that the proportion of fracture cases was almost equivalent to the normal incidence (40–50%) across different cohorts. This is particularly important, considering that a DL model trained on an imbalanced training set is more vulnerable to biases and more likely makes decisions in favor of the majority class^22^. Furthermore, the diagnostic performance of our DL model was compared to that of radiologists using a geographically separate dataset, which increases the generalizability of our results. Additionally, we applied Grad-CAM to overlay heatmaps onto radiographs to improve the transparency and interpretability of our DL model. In fact, the intensities of the heatmaps mostly focused on the nasal areas, suggesting that the model correctly recognized the nasal area when identifying a fracture, even in the presence of artifacts such as skin folds or metallic wires of facial masks.

There are, however, several limitations of the current study that need to be addressed. First, the sample size of the external test set was relatively small, which may somewhat reduce the generalizability of our DL model to real-world practice settings. Second, the number of reviewers was limited to two experienced radiologists. The potential benefit of the DL model could have been further validated if additional reviewers from different backgrounds, such as emergency physicians or plastic surgeons, had been included. Third, there was a significant difference in the proportion of sexes between training/validation and test sets. However, no significant anatomical difference in nasal bones has been found between sexes^23^, and thus this difference would have not affected the outcome of this study. Finally, the study cohorts comprised an Asian population whose nasal bone anatomy may differ from that of other ethnicities. Thus, the developed DL model may not yield similar results for patients of different ethnicities.

In conclusion, our CNN-based DL model demonstrated excellent performance comparable to that of experienced radiologists in diagnosing nasal bone fractures on conventional radiographs. This promising finding could translate into DL applications that can be used as diagnostic assistance tools for patients with facial trauma.

## Methods

The institutional review boards of Seoul St. Mary’s Hospital (KC22RISI0472) and Yeouido St. Mary’s Hospital (SC22RISI0102) approved this retrospective study and waived the requirement for informed consent considering the retrospective nature of the analyses involving only anonymized data. All methods were performed in accordance with the ethical standards of our institutional research committee and with the 1964 Helsinki declaration and its later amendments.

### Dataset

**Figure 4:**
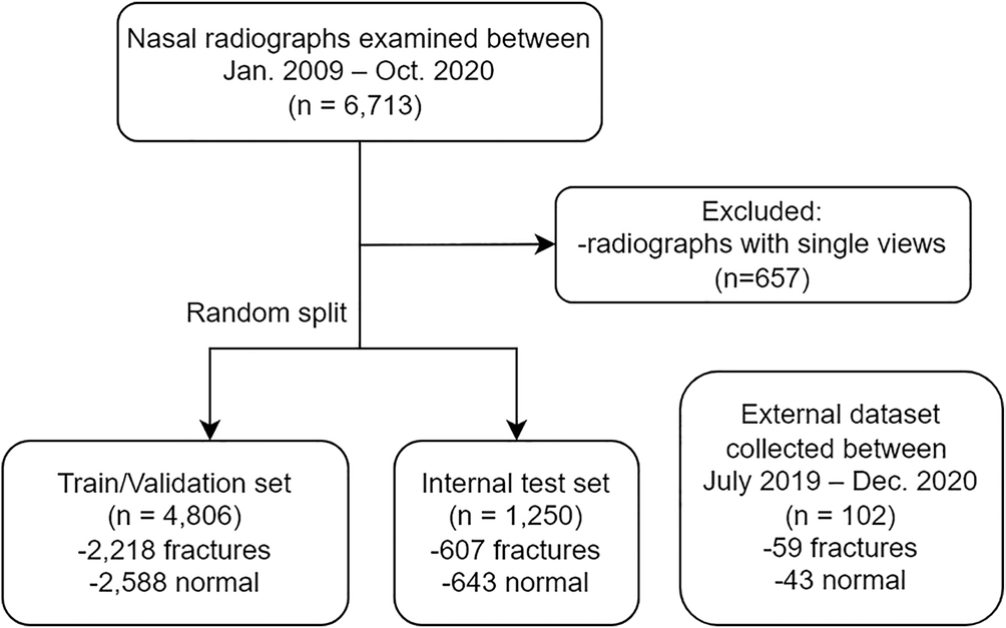
A flowchart of patient selection process.

A total of 9596 nasal radiographs from 6713 adult patients were examined for suspected nasal bone fractures between January 2009 and October 2020 at our institution. All nasal bone radiographs were exported from the institutional picture archiving and communication system in anonymized DICOM format. The data were exported and initially reviewed by a (blinded) 3rd-year radiology resident in training. The criteria for determining nasal bone fractures were based on surgical findings when available or clinical/radiological consensus obtained from electronic medical records. Nasal radiographs were examined bilaterally, and records of patients with only unilateral (left or right) nasal radiographs were excluded (n = 657). The remaining patients were then randomly divided into the training, validation, and internal test cohorts. The external test cohort consisted of patients who presented at a geographically separated tertiary hospital between July 2019 and December 2020 (Fig. 4).

### Image assessment by experienced radiologists

Two fellowship-trained radiologists (both blinded, with 6 and 9 years of experience in head and neck diagnostic radiology, respectively) independently reviewed and classified the nasal radiographs as either fractures or normal bone. Both radiologists routinely interpret nasal bone radiographs. A fracture was diagnosed if any radiologic features of nasal bone fractures, including fracture lines, displacement, depression, deformity, and angulation, were present.

### Image preprocessing

Before the DL model training, all images were preprocessed in the following way. First, images were resized by adjusting the image ratio, with the length of the short axis set to 512 pixels. Intensity normalization was then performed to obtain a pixel value between 0 and 1.

### Training the deep learning algorithm

**Figure 5:**
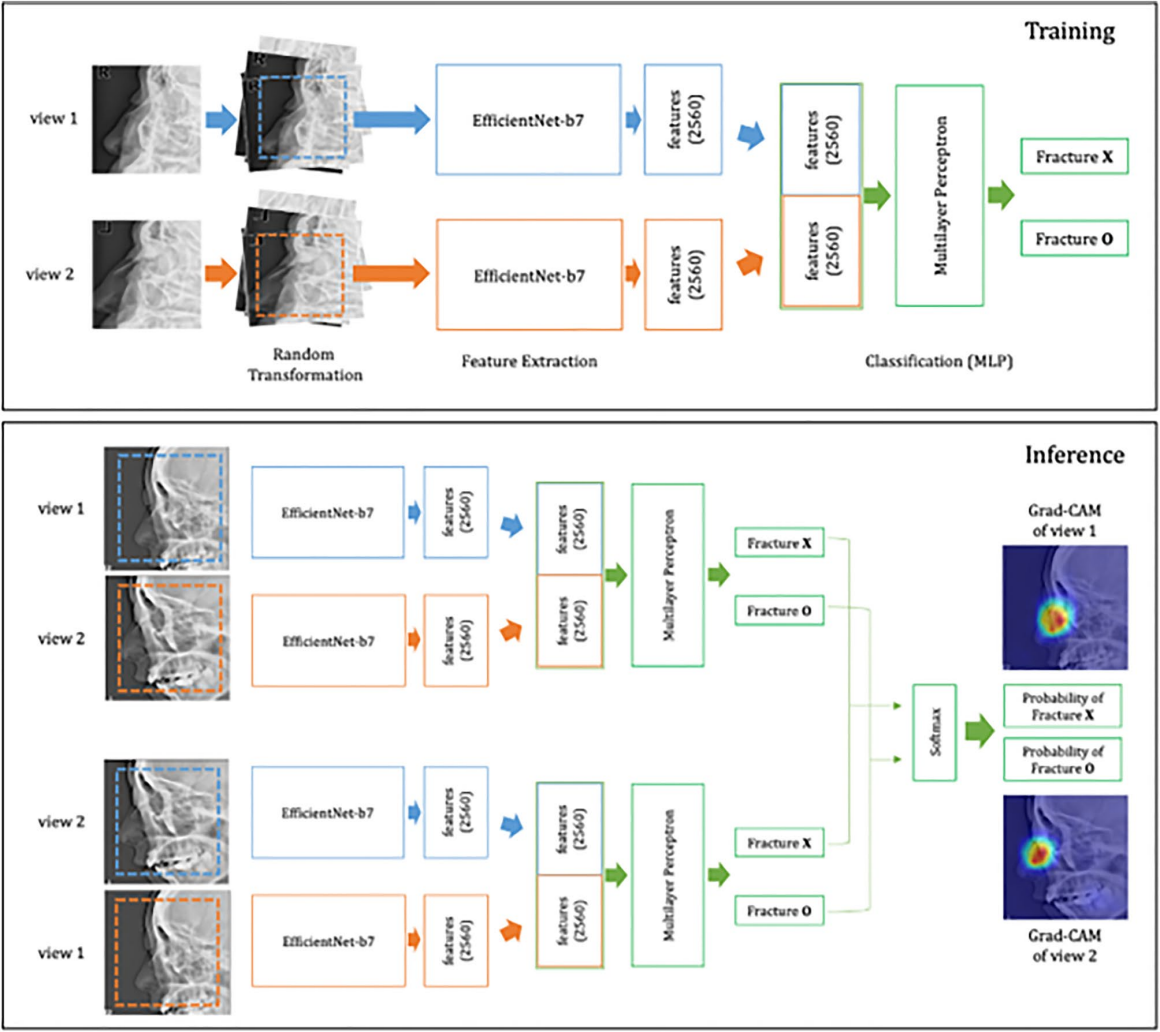
A workflow of training deep learning algorithm.

WE trained a DL model to classify the nasal radiographs. Two lateral views from each patient were used as the model input, and the presence of a nasal fracture was determined via binary classification. First, to simultaneously utilize the features of both views, the imaging features were independently extracted from each view using the backbone of EfficientNet-B7 model^24^. The parameters of the backbone were initialized by loading an ImageNet pretrained model. The input image size of each CNN model was 448 × 448 pixels, and 2560 imaging features were extracted. Then, binary classification was performed with a multilayer perceptron model using the concatenated 5120 features extracted from the two CNN model paths as inputs. The multilayer perceptron model consists of three hidden layers to reduce the number of hidden units by half for each hidden layer. To reduce overfitting of the training data, various random transformations, including random flipping, rotation, affine transforms, intensity inverting, addition of random noises, and random cropping were applied during the model training. Cross-entropy was used as a cost function, and the model parameters were updated using the AdamW algorithm^25^ with a learning rate of 0.0001 and a weight decay coefficient of 0.001. A cut-off value of 0.5 was used in the cross-entropy loss function such that the parameters were updated based on whether the prediction reached the cut-off value. For each epoch, the AUC for the validation set was calculated and the parameters in the epoch with the best AUC result (the 140th epoch out of the total of 256 epochs in our training process) were selected. After the training process was completed, in the inference of the test set, the probability of an image containing a nasal fracture was determined by averaging the two results, with the order of the two views changed. To identify the key regions that contributed to the model’s decision, Gradient-weighted Class Activation Mapping (Grad-CAM) was applied at the last convolutional layer of the CNN model for each view. We reviewed the Grad-CAM results of the external dataset to observe whether Grad-CAM properly emphasized the nasal bone area. The overall DL model architecture used in this study is summarized in Fig. 5, and more details and the codes of the model were uploaded to the Github repository (https://github.com/hufsaim/nasalbone).

### Statistical analysis

Continuous and categorical variables were compared among the training, validation, and internal/external test cohorts using one-way ANOVA and Pearson’s Chi-squared test, respectively. Cohen’s κ was calculated to assess the inter-rater agreement between the DL model and the two radiologists. The level of agreement was determined as none to slight if κ was 0.01–0.20; fair at κ = 0.21–0.40; moderate at 0.41–0.60; substantial at 0.61–0.80; and almost perfect at 0.81–1.00^26^. To evaluate the diagnostic performance of the DL model, the AUC was calculated for both the internal and the external test set. The optimal threshold for the ROC curve was determined using the Youden index. The AUCs of the DL model and the radiologists were compared using the DeLong method^27^. Confidence intervals for sensitivities, specificities, and accuracies were derived from 2000 bootstrap replicates using the “pROC” R package. All statistical analyses were performed using R statistical software (v. 4.1.2, Vienna, Austria) and Stata (v. 16.1, College Station, TX, USA). A two-sided *P*-value of less than 0.05 was considered statistically significant.

## Data Availability

The datasets used and/or analysed during the current study available from the corresponding author on reasonable request.

## Abbreviations

CNN: Convolutional neural network
DL: Deep learning
Grad-CAM: Gradient-weighted Class Activation Mapping
